# Higher serum lipocalin 2 is associated with post-stroke depression at discharge

**DOI:** 10.1186/s12883-023-03319-y

**Published:** 2023-08-05

**Authors:** Yufeng Liu, Lu Liu, Zhongwen Zhi, Rui Chen, Qing Wang, Mengchao Wang, Yuqian Wang, Liandong Zhao

**Affiliations:** Department of Neurology, Affiliated Huai’an Hospital of Xuzhou Medical University, No.62, South Huaihai Road, Huai’an 223002, Huai’an, 223002 Jiangsu China

**Keywords:** Acute ischemic stroke, Post-stroke depression, Inflammatory markers, Lipocalin 2, LCN2

## Abstract

**Background and aims:**

Post-stroke depression (PSD), as one of the common complications after stroke, seriously affects the physical and mental health and functional prognosis of patients. Previous studies have shown that the increase of inflammatory mediators is associated with the occurrence of PSD. Lipocalin 2 (LCN2), as an acute phase protein, is involved in the development of acute ischemic stroke (AIS), and its expression is up-regulated in patients with depression, suggesting that there is a potential correlation between serum LCN2 and depression. The aim of this study was to explore the relationship between serum LCN2 at admission and PSD at discharge.

**Methods:**

A total of 358 AIS patients were retrospectively included. All patients had fasting venous blood taken within 24 h of admission to detect serum LCN2. The patients were evaluated by 17-item Hamilton Depression Scale (HAMD) before discharge. Patients with HAMD score > 7 were diagnosed with PSD. The correlation between serum LCN2 and PSD was tested using binary logistic regression analysis.

**Results:**

In our study, 92 (25.7%) patients were diagnosed with PSD at discharge. According to the serum LCN2 value, the patients were divided into three layers (Tertile1 ≤ 105.24ng/ml; Tertile2: 105.24-140.12ng/ml; Tertile3 ≥ 140.12ng/ml), with T1 layer (the lowest levels) as a reference, after adjusting for multiple potential confounding factors, T3 layer (the highest levels) was independently associated with the occurrence of PSD (odds ratio [OR] = 2.639, 95% confidence interval [CI]: 1.317–5.287, *P* = 0.006). Similar results were found when the serum LCN2 was analyzed as a continuous variable. The optimal cut-off value of serum LCN2 at admission to predict PSD at discharge was 117.60ng/ml, at this threshold, the sensitivity was 77.2%, and the specificity was 53.4%.

**Conclusions:**

High serum LCN2 levels at admission are an independent risk factor for PSD in patients with AIS at discharge.

**Supplementary Information:**

The online version contains supplementary material available at 10.1186/s12883-023-03319-y.

## Introduction

 Acute ischemic stroke (AIS) accounts for 60-80% of all strokes, and its incidence has increased significantly [1]. It is characterized by high disability, high mortality, high recurrence rate, and many complications. Post-stroke depression (PSD), as one of the common complications and the most serious neuropsychiatric sequelae after stroke, severely affects the functional prognosis and quality of life of patients. Studies have shown that the incidence of PSD fluctuates between 18% and 33% [2]. Studies have reported that the occurrence of early PSD is an independent risk factor for adverse outcomes 5 years after stroke [3]. Stroke survivors with depression have higher suicidal ideation and all-cause mortality [4, 5]. Therefore, early identification of PSD is particularly crucial, and timely intervention can improve patients’ psychiatric symptoms and clinical outcomes.                                                       

The pathogenesis of PSD is complex and involves a combination of stroke and depression pathogenesis, which has not been thoroughly elucidated. The existing evidence supports that neurotransmitters, neuroinflammation, neuroendocrine and neuroplastic mechanisms play a role in the occurrence and development of PSD [6]. After stroke, the up-regulated expression of inflammatory mediators is associated with poor function and clinical prognosis [7]. There is increasing evidence that inflammation is involved in the etiology of depression through inflammatory pathways and inflammatory mediators. Numerous studies have explored the expression of inflammatory mediators in patients with PSD. The increase of pro-inflammatory cytokines such as interleukin (IL)-1β, IL-6, IL-18 and TNF-α is involved in the inflammatory response of depression [8, 9].

Lipocalin 2 (LCN2), also known as neutrophil gelatinase-associated lipocalin (NGAL), is a pleiotropic mediator of various inflammatory processes. It was originally isolated from neutrophils released from human infection and inflammatory sites [10]. In addition to being produced by activated neutrophils, it is expressed in adipocytes, endothelial cells, macrophages and renal tubular cells. LCN2 is involved in a variety of physiological and pathological processes such as cell differentiation, inflammation and immune response, apoptosis, signal transduction, glycolipid metabolism, tumor formation, invasion and metastasis [11]. LCN2 is thought to be involved in the development of AIS and to play an essential role in ischemia-reperfusion injury following AIS [12]. Extensive studies have found that after ischemic stroke, LCN2 is not only up-regulated in the central nervous system (CNS), but also significantly increased in serum [13]. As an inflammatory mediator, serum LCN2 is considered a valuable biomarker for clinical outcome in patients with AIS [14]. Peripheral inflammatory injury not only increases the expression and production of LCN2 in the cerebrovascular system, but also leads to the release of this molecule into the CNS [15]. Additional evidence also supports that LCN2 can cross the blood-brain barrier (BBB) [16]. Otherwise, serum LCN2 expression increased in patients with depression and was associated with depression score [17, 18]. In the animal experimental model, the serum LCN2 concentration of PSD mice was significantly increased and the expression of LCN2 mRNA in the hippocampus was significantly up-regulated at 1–3 weeks after surgery, compared to the stroke control group [19]. This suggests that LCN2 may be an essential etiological factor in PSD.

In summary, serum LCN2 is up-regulated after ischemic stroke and LCN2 is strongly associated with depression. Currently, there are no studies on the relationship between serum LCN2 and PSD in human samples. This study aims to explore the relationship between serum LCN2 and PSD in patients with AIS and its clinical application value.

## Materials and methods

### Study population

This study retrospectively included AIS patients hospitalized in the Department of Neurology, Affiliated Huai’an Hospital of Xuzhou Medical University from January 2022 to February 2023.

**Fig. 1:**
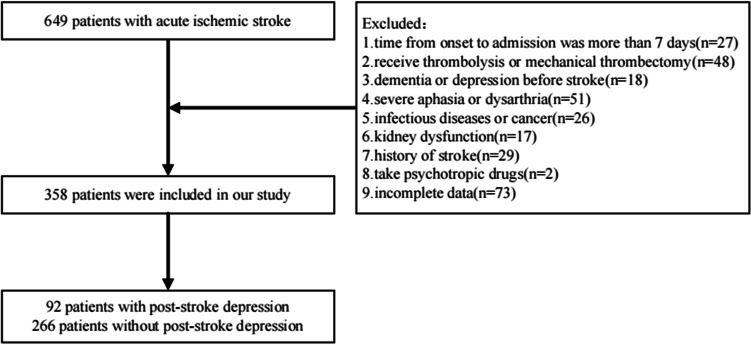
Included and excluded patients

Inclusion criteria: (a) diagnosed as AIS and confirmed by computed tomography or magnetic resonance imaging at admission; (b) The onset time was within 7 days; (c) Between 18 and 80 years old; (d) Stroke was involved for the first time; (e) Not receiving thrombolytic therapy and endovascular treatment; (f) Complete clinical data. Exclusion criteria: (a) Obvious cognitive impairment before stroke or combined with CNS diseases such as Parkinson ‘s disease; (b) Combined with severe dysarthria or aphasia, can not cooperate to complete the relevant scale assessment; (c) A history of mental illness including depression before stroke; (d) Taking antidepressants and other psychiatric drugs; (e) Renal insufficiency (estimated glomerular filtration rate < 60ml/min/1.73m^2^); (f) Severe liver dysfunction, pulmonary dysfunction, acute and chronic inflammatory diseases and other acute vascular ischemic diseases including acute myocardial infarction; (g) Previous autoimmune diseases, malignant tumors and hematological diseases. Ultimately, a total of 358 participants met the study criteria and had their data analyzed, of whom 92 were diagnosed with PSD at discharge and 266 were not. The incidence of PSD in this study was 25.7% (Fig. 1).

The study protocol was approved by the Ethics Committee of Affiliated Huai’an Hospital of Xuzhou Medical University (Approval No. HEYLL202229 of Institutional Review Committee). This research is based on the principles of the Helsinki Declaration. Patients included in the study were informed of the study and signed informed consent. For illiterate patients, we obtained the informed consent of their legal guardian.

### Collection of baseline data

The general data of patients were collected through the hospital database. Including hospitalization days, demographic information [gender, age, education, marital status and body mass index (BMI)], vascular risk factors (hypertension, diabetes, coronary heart disease, hyperlipidemia, smoking and drinking), neuropsychological assessment [National Institutes of Health Stroke Scale (NIHSS) [20] at admission was used to record the degree of neurological deficit, Barthel Index (BI) [21] and modified Rankin Scale (mRS) [22] were used to evaluate the functional prognosis of patients at discharge, and 17-item Hamilton Depression Scale (HAMD) [23] was used to evaluate the mental health of patients before discharge (day 7–14 after the onset of stroke)]. Stroke subtypes classified according to the criteria of the Trial of Org 10,172 in Acute Stroke Treatment (TOAST) [24] were also collected. In addition, we collected the volume of cerebral infarction in the patients. The volume of cerebral infarction (according to the Pullicino formula) [25] = the longest diameter × the widest diameter × the number of layers × (layer spacing + layer thickness) × 0.5 shown on head computed tomography/magnetic resonance imaging.

### Psychological measurement

All patients were assessed with the 17-item Hamilton Depression Scale (HAMD) [23] before discharge (day 7–14 after the onset of stroke). According to Structured Clinical Interview for the Diagnostic and Statistical Manual of Mental Disorders, 4th edition. Patients with HAMD score > 7 were diagnosed with PSD. Doctors who assessed the scale were not aware of the clinical symptoms and laboratory data of the patients.

### Laboratory data testing

Peripheral venous blood was collected from all patients within 24 h of admission and fasting for more than 8 h for detection, including serum LCN2, leucocyte, neutrophil, lymphocyte, high-sensitivity C-reactive protein (hs-CRP) and serum creatinine (Scr). Estimate glomerular filtration rate (eGFR) was calculated according to serum creatinine level, age and gender. The eGFR was calculated using a Chinese population-adjusted formula [26], eGFR(ml/min/1.73m^2^) = 175×[Scr(mg/dl)]^-1.234^×[age(years)]^-0.179^×gender(male = 1, female = 0.79).

Detection of LCN2: After standing for 30 min, the eligible blood sample was centrifuged at 3500 r/min for 15 min and the serum was separated to ensure that the sample was free of haemolysis. LCN2 level was detected by magnetic particle chemiluminescence immunoassay (double antibody sandwich method). LCN2 reagent was purchased from Beijing Hotgen Biotech Co.,Ltd.

### Statistical analysis

For this study, all statistical analyses were performed using SPSS version 25.0 (SPSS Inc., Chicago, IL, USA). Normally distributed data are expressed as the mean ± standard deviation (SD), and group comparisons were performed by two independent-sample t tests. Nonnormally distributed data are expressed as the median and interquartile range. Group comparisons were made with the Mann‒Whitney U test. Count data are expressed as the number and percentage (n,%), and group comparisons were performed using the chi-square test. The relationship between serum LCN2 and PSD was tested by univariate and multivariate binary logistic regression analysis. Correlation analyses were performed using Pearson correlation or Spearman rank-order correlation. A receiver-operating characteristic (ROC) curve was used to estimate the optimal cut-off value of serum LCN2 for predicting PSD in patients with AIS, and the area under the curve (AUC), sensitivity and specificity of the ROC were evaluated. *P* < 0.05 was considered statistically significant.

## Results

### Comparison of baseline data between PSD and Non-PSD groups

In the baseline data of the two groups of patients shown in Table 1, the PSD group was mainly female (62.0% vs. 41.0%, *P* = 0.001) and had a lower years of education (3 (0, 6) vs. 5 (2, 6), *P* = 0.020). We found significant differences in serum LCN2 (139.90 (118.80, 178.85) vs. 113.95 (87.99, 143.63), *P* < 0.001) and eGFR (98.17 (81.32, 119.45) vs. 112.38 (89.03, 138.97), *P* = 0.001) between the two groups. Compared with non-PSD group, patients in PSD group had more severe stroke symptoms, higher NIHSS score at admission (3 (2, 5) vs. 2 (1, 4), *P* = 0.008), higher mRS score at discharge (1 (1, 3) vs. 1 (1, 2), *P* = 0.006) and lower BI score (85 (70, 90) vs. 90 (80, 100), *P* = 0.010). In addition, patients with PSD had higher cerebral infarct volumes than those without PSD (2297.50 (993.10, 5289.86) vs. 1794.60 (760.80, 3645.08), *P* = 0.029). There was no significant difference in age, marital status, BMI, hospitalization days, hypertension, diabetes, coronary heart disease, hyperlipidemia, smoking, drinking, cause of stroke, leucocyte, neutrophil, lymphocyte and high-sensitivity C-reactive protein between the two groups (*P* > 0.05) (Table 1).

**Table 1:** Comparison of baseline data between PSD group and Non-PSD group

Variables	All patients (*n* = 358)	PSD group (*n* = 92)	Non-PSD group (*n* = 266)	*P* value
**Demographic data**				
Age, years	65 (59, 70)	66 (60, 71)	64 (59, 70)	0.169
Female (%)	166 (46.4)	57 (62.0)	109 (41.0)	0.001
Education, years	4 (1, 6)	3 (0, 6)	5 (2, 6)	0.020
Married (%)	348 (97.2)	89 (96.7)	259 (97.4)	0.752
BMI, kg/m²	24.77 (23.22, 26.87)	24.50 (23.08, 26.67)	24.87 (23.23, 26.92)	0.789
Hospitalization days, days	12 (10, 14)	13 (10, 15)	12 (10, 14)	0.205
**Vascular risk factors**				
Hypertension (%)	280 (78.2)	71 (77.2)	209 (78.6)	0.780
Diabetes (%)	104 (29.1)	24 (26.1)	80 (30.1)	0.468
Coronary heart disease (%)	62 (17.3)	18 (19.6)	44 (16.5)	0.509
Hyperlipidemian (%)	111 (31.0)	27 (29.3)	84 (31.6)	0.690
Smoking (%)	106 (29.6)	25 (27.2)	81 (30.5)	0.553
Drinking (%)	66 (18.4)	16 (17.4)	50 (18.8)	0.764
**Stroke subtypes**				0.592
LAA (%)	271 (75.7)	73 (79.3)	198 (74.4)	
CE (%)	42 (11.7)	8 (8.7)	34 (12.8)	
SAO (%)	33 (9.2)	7 (7.6)	26 (9.8)	
ESUS (%)	12 (3.4)	4 (4.3)	8 (3.0)	
**Neuropsychological function**				
NIHSS on admission, score	2.5 (1, 4)	3 (2, 5)	2 (1, 4)	0.008
BI score at discharge, score	90 (80, 95)	85 (70, 90)	90 (80, 100)	0.010
mRS score at discharge, score	1 (1, 2)	1 (1, 3)	1 (1, 2)	0.006
HAMD score, score	3 (2, 8)	12 (9, 15)	2 (1, 4)	<0.001
**Laboratory data**				
Leucocyte, ×10^9^ /L	6.91 (5.67, 8.30)	6.90 (6.08, 8.41)	6.95 (5.53, 8.30)	0.233
Neutrophil, ×10^9^ /L	4.72 (3.51, 5.76)	4.82 (4.06, 5.66)	4.64 (3.32, 5.78)	0.085
Lymphocyte, ×10^9^ /L	1.58 (1.23, 2.03)	1.55 (1.23, 1.89)	1.59 (1.22, 2.14)	0.305
Hs-CRP, mg/L	2.57 (1.05, 3.56)	2.68 (1.61, 3.95)	2.57 (0.90, 3.49)	0.192
LCN2, ng/ml	121.91 (93.13, 155.20)	139.90 (118.80, 178.85)	113.95 (87.99, 143.63)	<0.001
eGFR, ml/min/1.73m^2^	107.82 (87.00, 133.97)	98.17 (81.32, 119.45)	112.38 (89.03, 138.97)	0.001
**Infarct volume, mm** ^**3**^	1849.88 (822.79, 4125.00)	2297.50 (993.10, 5289.86)	1794.60 (760.80, 3645.08)	0.029

*Abbreviations*: *PSD* post-stroke depression, *Non-PSD* Non-post-stroke depression, *BMI* body mass index, *LAA* Large artery atherosclerosis, *CE* Cardioembolism, *SAO* small-artery occlusion, *ESUS* embolic stroke of undetermined source, *NIHSS* National Institutes of Health Stroke Scale, *BI* Barthel Index, *mRS* modified Rankin Scale, *HAMD* Hamilton Depression Scale, *Hs-CRP* High-sensitivity C-reactive protein, *LCN2* Lipocalin 2, *eGFR* estimated glomerular filtration rate.

### The relationship between serum LCN2 and PSD

**Fig. 2:**
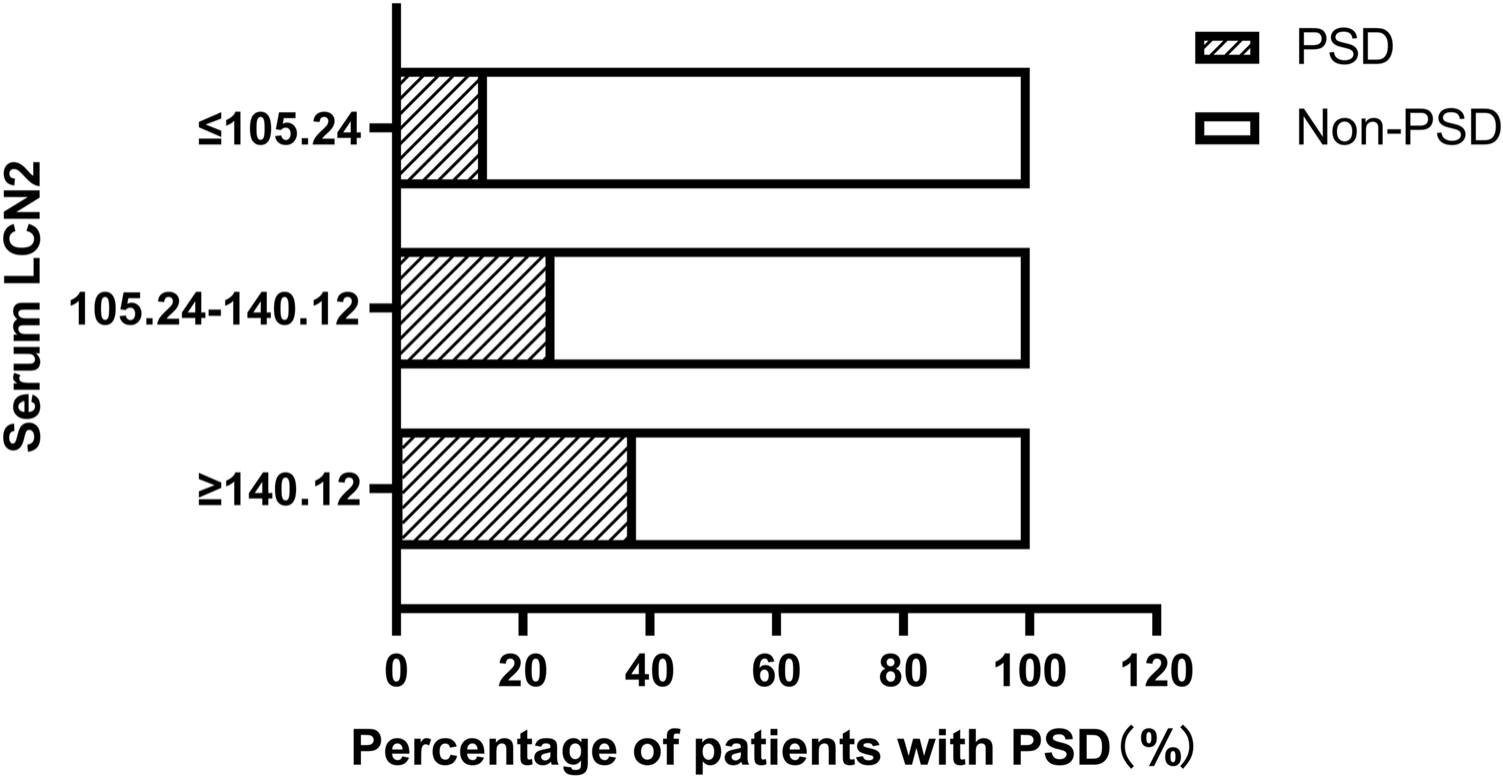
Relationship between serum LCN2 and PSD incidence. Abbreviations: PSD post-stroke depression, Non-PSD Non-post-stroke depression, LCN2 Lipocalin 2

We observed a significant difference in serum LCN2 between PSD and non-PSD patients. In order to further explore the relationship between serum LCN2 and PSD, we divided the patients into three groups according to the serum LCN2 value (Tertile1 LCN2 ≤ 105.24ng/ml, n = 119; Tertile2 LCN2: 105.24-140.12ng/ml, n = 120; Tertile3 serum LCN2 ≥ 140.12ng/ml, n = 119). In Fig. 2, the incidence of PSD increased with the increase of serum LCN2 level (Tertile1 14.3%; Tertile2 25.0%; Tertile3 37.8%). Chi-square test showed that there was a significant difference in the proportion of PSD and non-PSD patients (*P* < 0.001).

Correlation analysis showed that serum LCN2 level was positively correlated with HAMD score in the total participants (r = 0.290, *P* < 0.001). This correlation was mainly found in PSD participants (r = 0.437, *P* < 0.001), but not in non-PSD participants (r = 0.102, *P* = 0.098).

**Fig. 3:**
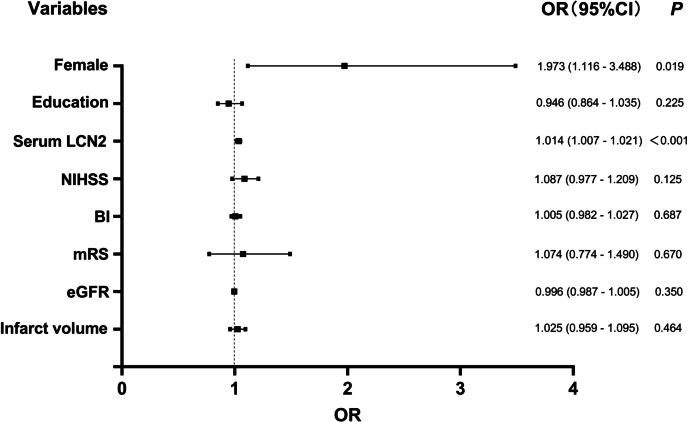
Forest plot of odds ratios for PSD. Abbreviations: PSD post-stroke depression, LCN2 Lipocalin 2, NIHSS National Institutes of Health Stroke Scale, BI Barthel Index, mRS modified Rankin Scale, eGFR estimated glomerular filtration rate. In this step, the unit of infarct volume is cm^3^

In the multivariate regression analysis, as shown in Table 2, with the occurrence of PSD as the dependent variable and the T1 layer of serum LCN2 as a reference, univariate logistic regression analysis showed that individuals in T2 layer (odds ratio [OR] = 2.000, 95% confidence interval [CI]: 1.035–3.866, *P* = 0.039) and T3 layer (OR = 3.649, 95% CI: 1.937–6.873, *P* < 0.001) had a higher risk of PSD. After adjusting for gender, education, NIHSS score, BI score, mRS score, eGFR and infarct volume, T3 layer was independently associated with the occurrence of PSD (OR = 2.639, 95% CI: 1.317–5.287, *P* = 0.006), while T2 layer was not significantly associated with the occurrence of PSD (OR = 1.646, 95% CI: 0.817–3.317, *P* = 0.163) (Table 2). As shown in Fig. 3 forest plot, when serum LCN2 was used as a continuous variable, serum LCN2 was still associated with PSD in logistic regression analysis, and this correlation was still significant even after confounding factors were controlled (unadjusted: OR = 1.017, 95% CI: 1.011–1.023, *P* < 0.001; after adjustment: OR = 1.014, 95% CI: 1.007–1.021, *P* < 0.001) (Fig. 3). In addition, Fig. 3 also showed that female (OR = 1.973, 95% CI: 1.116–3.488, *P* = 0.019) was also independently associated with an increased risk of PSD (Fig. 3).

**Fig. 4:**
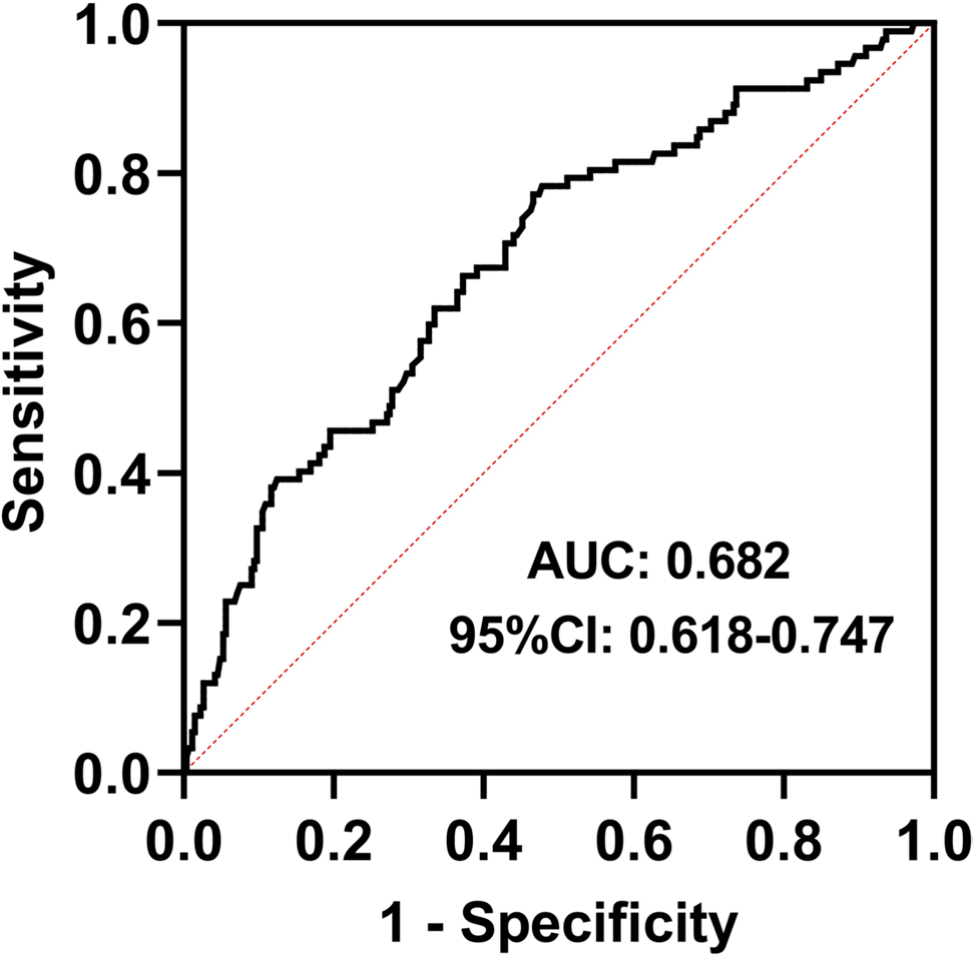
ROC curve of serum LCN2 level and PSD

According to the ROC curve (Fig. 4), the optimal cut-off value of serum LCN2 for predicting PSD was 117.60ng/ml, and the Youden index was 0.3055. The corresponding sensitivity was 77.2%, specificity was 53.4%, and AUC was 0.682 (95% CI: 0.618–0.747).

**Table 2:** Multivariate adjusted odds ratios for the association between serum LCN2 and PSD

	Tertiles	OR	95%CI	*P* value
Unadjusted	Tertile1	reference		
	Tertile2	2.000	1.035–3.866	0.039
	Tertile3	3.649	1.937–6.873	<0.001
Model 1	Tertile1	reference		
	Tertile2	1.796	0.903–3.574	0.095
	Tertile3	2.932	1.489–5.773	0.002
Model 2	Tertile1	reference		
	Tertile2	1.646	0.817–3.317	0.163
	Tertile3	2.639	1.317–5.287	0.006

Model 1: adjusted for gender, education, NIHSS score, BI score, mRS score and infarct volume

Model 2: adjusted for covariates from model 1 and further adjusted for eGFR

### Correlation analysis between serum LCN2 and NIHSS score, cerebral infarction volume

**Fig. 5:**
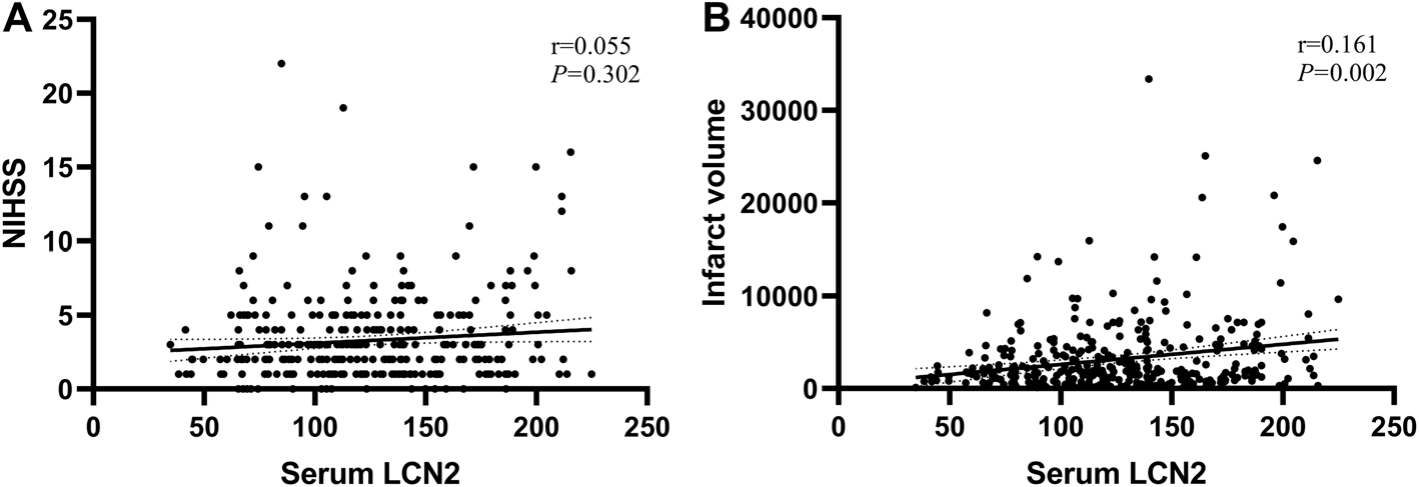
Correlation analysis between serum LCN2 and NIHSS score, cerebral infarction volume. Abbreviations: *LCN2* Lipocalin 2, *NIHSS* National Institutes of Health Stroke Scale

In Spearman correlation analysis, serum LCN2 was not significantly correlated with NIHSS score (*P* > 0.05) (Fig. 5A), while serum LCN2 was positively correlated with cerebral infarction volume (*r* = 0.161, *P* = 0.002) (Fig. 5B).

## Discussion

The incidence of PSD at discharge was 25.7% in our study, and baseline serum LCN2 levels were significantly higher in PSD patients than in non-PSD patients. Further multivariate logistic regression analysis showed that compared with lower serum LCN2, even after adjusting for confounding factors such as gender, education, NIHSS score, BI score, mRS score, eGFR and infarct volume, higher serum LCN2 at admission was still independently associated with the occurrence of PSD at discharge. Individuals in T3 layer had a 2.639 times higher probability of PSD at discharge than those in T1 layer (OR = 2.639, 95% CI: 1.317–5.287, *P* = 0.006). According to the ROC curve, the optimal cut-off value of serum LCN2 at admission to predict PSD at discharge was 117.60ng/ml, with a sensitivity of 77.2% and a specificity of 53.4%. Previous studies have shown that women are independent risk factors for PSD [27], including in other types of depression, women tend to be more susceptible to depression, which is consistent with our findings (OR = 1.973, 95% CI: 1.116–3.488, *P* = 0.019).

It is reported that depression has become the main cause of disability [28]. Stroke patients are usually associated with depression, which commonly leads to deterioration of the patients’ condition and slow recovery [2]. Therefore, it is necessary to explore the pathogenesis of PSD and PSD-related body fluid markers. As a heterogeneous disease, the pathogenesis of PSD is complex and involves brain injury and psychological stress caused by various pathologies. A single pathophysiological mechanism is not sufficient for a complete explanation. Some processes that may lead to PSD mainly include decreased monoamine levels, abnormal neurotrophic response to stroke, increased inflammation and hypothalamic-pituitary-adrenal axis disorders and glutamate-mediated excitatory toxicity [29]. Extensive studies have shown that neuroinflammation caused by stroke may play an essential role in the development of PSD [30, 31].

As a member of the lipid carrier protein family, LCN2 has various functions such as inflammatory response, insulin sensitivity, cell differentiation and cell migration [32]. LCN2 in the peripheral circulation can be used as a biomarker for numerous diseases, such as metabolic syndrome, acute kidney injury, cardiovascular disease or various brain diseases [33]. When stroke occurs, reactive astrocytes, activated microglia, neurons, choroid plexus and endothelial cells synthesize and secrete LCN2 in response to inflammation or damage to the brain [34]. It is reported that elevated levels of LCN2 in human plasma 1–3 days after ischaemic stroke [35, 36], and plasma LCN2 still has a high expression level in the subacute phase [37]. Significantly elevated LCN2 in cerebrospinal fluid (CSF) or peripheral blood is associated with poor functional prognosis after stroke [14]. Previous studies have suggested that plasma LCN2 is involved in the early inflammatory events of mild cognitive impairment (MCI) and Alzheimer ‘s disease (AD). Elevated plasma LCN2 levels may reflect CNS inflammation in patients with MCI and AD [38]. Olson B et al. [39] observed that under the condition of ensuring the integrity of BBB, LCN2 in CSF changed the same after the increase of LCN2 in peripheral blood, which means that LCN2 can easily cross BBB. Based on the above research, we believe that serum LCN2 can reflect the neuroinflammatory state of patients to a certain extent.

LCN2 has been shown to be associated with multiple biological behavioral activities such as pain hypersensitivity, cognitive function, emotion, depression and anxiety [34]. Naudé PJ et al. [40] observed that the plasma LCN2 level in patients with depression was significantly higher than that in the non-depression control group. Compared with patients with the first episode, the plasma LCN2 level in patients with recurrent depression was higher. LCN2 is considered to be a potential diagnostic biomarker for depression. Another study by the team showed that depression in patients with chronic heart failure was associated with elevated plasma LCN2, but not with the clinical severity of the underlying disease [18]. The relationship between LCN2 and depression should not be ignored. Therefore, our study explored the association between serum LCN2 at admission and PSD at discharge for the first time in human samples. The results suggest that patients with depression have higher serum LCN2 levels. After adjusting for multiple confounding factors, high baseline serum LCN2 is an independent risk factor for PSD at discharge, and it showed significant diagnostic accuracy in distinguishing PSD patients from patients without depression.

Serum LCN2 is often widely used as an indicator of renal injury [41]. Our data suggest that elevated serum LCN2 is associated with lower eGFR. Baseline data showed that PSD patients had lower eGFR levels than non-PSD patients. When multivariate binary logistic regression analysis was included, the correlation between renal function and PSD was not significant (OR = 0.996, 95% CI: 0.987–1.005, *P* = 0.350). After stroke, an increase in serum LCN2 is not only caused by kidney injury, but may also originate in the CNS. Based on the current data, we cannot attribute the association between high serum LCN2 levels and PSD to impaired renal function. Naudé PJ et al. [18] also believed that elevated serum LCN2 levels in depressed patients with chronic heart failure may reflect inflammation associated with depressive symptoms, but not with the presence of renal dysfunction.

The exact biological mechanism behind the observed correlation between serum LCN2 and PSD remains unclear. Relevant basic studies have shown that after ischemic stroke, elevated LCN2 activates various inflammatory pathways in glial cells, leading to glial cell proliferation, cytokine production and enhanced inflammatory response, so that neuroinflammation persists [34]. The abnormal increase of LCN2 not only directly destroys the BBB, but also forms a complex with matrix metallopeptidase-9 (MMP-9), which prolongs the activity of MMP-9 and participates in the destruction of the BBB [37]. Moreover, peripheral neurotoxic substances that pass through the damaged BBB also exacerbate neuroinflammation. The application of LCN2 specific monoclonal antibody in the time window can significantly reduce the levels of LCN2 mRNA and protein and pro-inflammatory mediators, reduce neurological deficits, cerebral infarction, edema, BBB leakage and neutrophil infiltration, and improve the functional prognosis of stroke [42]. Mucha M et al. [43] suggested that LCN2 is an important regulator of stress-induced dendritic spine density and morphological changes in the hippocampus. Increased brain LCN2 levels may result in decreased hippocampal synaptic spine density. Wei L et al. [19] observed that hippocampal microglia were activated during PSD in PSD mouse model. LCN2 may regulate the activation of hippocampal microglia via the P38 MAPK pathway and be involved in the development of PSD. The findings of this study also found that patients with higher serum LCN2 levels had more severe depressive symptoms. Combined with the above studies, this suggests that LCN2 plays a crucial role in the process of PSD and that LCN2 can be used as a promising biomarker for PSD.

Our data showed a positive correlation between serum LCN2 and cerebral infarct volume (r = 0.161, *P* = 0.002). Unfortunately, we did not find a correlation between serum LCN2 and NIHSS score, which may be related to the fact that we excluded some patients with severe symptoms who were not able to cooperate with the scale assessment. In addition, we analyzed the differences in the expression of serum LCN2 levels between stroke subtypes, and patients in the large artery atherosclerosis group had significantly higher serum LCN2 levels than those in the small-artery occlusion group (see Additional file 1). Previous studies have shown a positive association between circulating LCN2 and carotid intima-media thickness and subclinical atherosclerosis in type 2 diabetes, suggesting a potential role of LCN2 as a member of the lipoprotein family in atherosclerosis pathogenesis [44]. This difference may be related to the severity of atherosclerosis. LCN2 is considered to be an inflammatory factor associated with aging and is associated with several CNS diseases [45]. Our data support a positive correlation of serum LCN2 with age even after the onset of AIS (r = 0.135, *P* = 0.010), but serum LCN2 did not differ significantly between genders (see Additional file 1).

Nevertheless, this study has several limitations. Firstly, due to our strict inclusion and exclusion criteria, we excluded patients with severe aphasia, dysarthria and renal insufficiency, which may lead to selection bias and underestimate the incidence of PSD. Secondly, we only measured serum LCN2 once within 24 h of admission and were not able to assess the relationship between dynamic changes in serum LCN2 and PSD. In addition, we only assessed depressive symptoms at discharge, while future studies could assess depressive symptoms and conduct longitudinal studies during long-term follow-up after stroke. Thirdly, we did not test the level of LCN2 in CSF. Simultaneous detection of CSF and serum LCN2 levels in patients will provide more insights for future research. Finally, we do not have detailed data on pre-stroke serum LCN2 and cannot establish causality based on observational data.

## Conclusions

In conclusion, high serum LCN2 levels at admission were independently associated with the occurrence of PSD in patients with AIS at discharge, which could provide a reference for the identification of early PSD in patients with AIS. Our findings should be considered preliminary, and further studies are needed to verify this association.

### Supplementary Information

**Additional file 1.** Relationship between serum LCN2 and gender, age. Relationship between serum LCN2 and Stroke subtypes.

## Data Availability

The datasets generated and analysed during the current study are not publicly available due to data protection of the patients and their relatives but are available from the corresponding author on reasonable request.

## Abbreviations

AIS: Acute ischemic stroke
PSD: Post-stroke depression
Non-PSD: Non-post-stroke depression
IL: Interleukin
LCN2: Lipocalin 2
NGAL: Neutrophil gelatinase-associated lipocalin
CNS: Central nervous system
BBB: Blood-brain barrier
BMI: Body mass index
NIHSS: National Institutes of Health Stroke Scale
BI: Barthel Index
mRS: modified Rankin Scale
HAMD: Hamilton Depression Scale
TOAST: The Trial of ORG 10172 in Acute Stroke Treatment
LAA: Large artery atherosclerosis
CE: Cardioembolism
SAO: Small-artery occlusion
ESUS: Embolic stroke of undetermined source
Scr: Serum creatinine
Hs-CRP: High-sensitivity C-reactive protein
eGFR: estimate glomerular filtration rate
ROC: Receiver operating characteristic
AUC: Area Under Curve
OR: Odds ratio
CI: Confidence interval
CSF: Cerebrospinal fluid
MCI: Mild cognitive impairment
AD: Alzheimer 's disease
MMP-9: Matrix metallopeptidase-9

## References

[1] Wu S, Wu B, Liu M (2019). Stroke in China: advances and challenges in epidemiology, prevention, and management. Lancet Neurol.

[2] Medeiros GC, Roy D, Kontos N (2020). Post-stroke depression: a 2020 updated review. Gen Hosp Psychiatry.

[3] Zeng YY, Wu MX, Geng DD (2021). Early-onset depression in stroke patients: effects on unfavorable outcome 5 years post-stroke. Front Psychiatry.

[4] Jørgensen TS, Wium-Andersen IK, Wium-Andersen MK (2016). Incidence of depression after stroke, and associated risk factors and mortality outcomes, in a large cohort of Danish patients. JAMA Psychiatry.

[5] Bartoli F, Pompili M, Lillia N (2017). Rates and correlates of suicidal ideation among stroke survivors: a meta-analysis. J Neurol Neurosurg Psychiatry.

[6] Ferrari F, Villa RF (2017). The neurobiology of depression: an integrated overview from biological theories to clinical evidence. Mol Neurobiol.

[7] McCabe JJ, Walsh C, Gorey S (2023). C-reactive protein, interleukin-6, and vascular recurrence after stroke: an individual participant data meta-analysis. Stroke.

[8] Kang HJ, Bae KY, Kim SW (2016). Effects of interleukin-6, interleukin-18, and statin use, evaluated at acute stroke, on post-stroke depression during 1-year follow-up. Psychoneuroendocrinology.

[9] Kim JM, Kang HJ, Kim JW (2017). Associations of tumor necrosis factor-α and interleukin-1β levels and polymorphisms with post-stroke depression. Am J Geriatr Psychiatry.

[10] Kjeldsen L, Johnsen AH, Sengeløv H (1993). Isolation and primary structure of NGAL, a novel protein associated with human neutrophil gelatinase. J Biol Chem.

[11] Rahimi S, Roushandeh AM, Ahmadzadeh E (2020). Implication and role of neutrophil gelatinase-associated lipocalin in cancer: lipocalin-2 as a potential novel emerging comprehensive therapeutic target for a variety of cancer types. Mol Biol Rep.

[12] Wang G, Weng YC, Han X (2015). Lipocalin-2 released in response to cerebral ischaemia mediates reperfusion injury in mice. J Cell Mol Med.

[13] Zhao RY, Wei PJ, Sun X (2023). Role of lipocalin 2 in stroke. Neurobiol Dis.

[14] Hochmeister S, Engel O, Adzemovic MZ (2016). Lipocalin-2 as an infection-related biomarker to predict clinical outcome in ischemic stroke. PLoS One.

[15] Olson B, Zhu X, Norgard MA (2021). Chronic cerebral lipocalin 2 exposure elicits hippocampal neuronal dysfunction and cognitive impairment. Brain Behav Immun.

[16] Mosialou I, Shikhel S, Liu JM (2017). MC4R-dependent suppression of appetite by bone-derived lipocalin 2. Nature.

[17] Naudé PJ, den Boer JA, Comijs HC (2014). Sex-specific associations between Neutrophil Gelatinase-Associated Lipocalin (NGAL) and cognitive domains in late-life depression. Psychoneuroendocrinology.

[18] Naudé PJ, Mommersteeg PM, Zijlstra WP (2014). Neutrophil gelatinase-associated lipocalin and depression in patients with chronic heart failure. Brain Behav Immun.

[19] Wei L, Du Y, Xie Y (2021). Lipocalin-2 regulates hippocampal microglial activation in poststroke depression. Front Aging Neurosci.

[20] Kwah LK, Diong J (2014). National Institutes of Health Stroke Scale (NIHSS). J Physiother.

[21] Quinn TJ, Langhorne P, Stott DJ (2011). Barthel index for stroke trials: development, properties, and application. Stroke.

[22] Broderick JP, Adeoye O, Elm J (2017). Evolution of the modified Rankin scale and its use in future stroke trials. Stroke.

[23] Spalletta G, Cravello L, Imperiale F (2013). Neuropsychiatric symptoms and interleukin-6 serum levels in acute stroke. J Neuropsychiatry Clin Neurosci.

[24] Adams HP, Bendixen BH, Kappelle LJ (1993). Classification of subtype of acute ischemic stroke. Definitions for use in a multicenter clinical trial. TOAST. Trial of Org 10172 in acute stroke treatment. Stroke.

[25] Hong G, Li T, Zhao H (2023). Diagnostic value and mechanism of plasma S100A1 protein in acute ischemic stroke: a prospective and observational study. PeerJ.

[26] Ma YC, Zuo L, Chen JH (2006). Modified glomerular filtration rate estimating equation for Chinese patients with chronic kidney disease. J Am Soc Nephrol.

[27] Zhu J, Wang L, Shao H (2022). Higher plasma fibrinogen level at admission is associated with post-stroke depression at discharge. Brain Sci.

[28] Friedrich MJ (2017). Depression is the leading cause of disability around the world. JAMA.

[29] Loubinoux I, Kronenberg G, Endres M (2012). Post-stroke depression: mechanisms, translation and therapy. J Cell Mol Med.

[30] Chen H, Liu F, Sun D (2022). The potential risk factors of early-onset post-stroke depression from immuno-inflammatory perspective. Front Immunol.

[31] Yang Y, Zhu L, Zhang B (2022). Higher levels of C-reactive protein in the acute phase of stroke indicate an increased risk for post-stroke depression: a systematic review and meta-analysis. Neurosci Biobehav Rev.

[32] Moschen AR, Adolph TE, Gerner RR (2017). Lipocalin-2: a master mediator of intestinal and metabolic inflammation. Trends Endocrinol Metab.

[33] Asaf S, Maqsood F, Jalil J, et al. Lipocalin 2-not only a biomarker: a study of current literature and systematic findings of ongoing clinical trials. Immunol Res. 2022:1–27. 10.1007/s12026-022-09352-2. 10.1007/s12026-022-09352-2 PMC9760530 36529828

[34] Jha MK, Lee S, Park DH (2015). Diverse functional roles of lipocalin-2 in the central nervous system. Neurosci Biobehav Rev.

[35] Elneihoum AM, Falke P, Axelsson L (1996). Leukocyte activation detected by increased plasma levels of inflammatory mediators in patients with ischemic cerebrovascular diseases. Stroke.

[36] Falke P, Elneihoum AM, Ohlsson K (2000). Leukocyte activation: relation to cardiovascular mortality after cerebrovascular ischemia. Cerebrovasc Dis.

[37] Zhang Y, Wang Y, Wu W (2022). Elevation of neutrophil carcinoembryonic antigen-related cell adhesion molecule 1 associated with multiple inflammatory mediators was related to different clinical stages in ischemic stroke patients. J Clin Lab Anal.

[38] Choi J, Lee HW, Suk K (2011). Increased plasma levels of lipocalin 2 in mild cognitive impairment. J Neurol Sci.

[39] Olson B, Zhu X, Norgard MA (2021). Lipocalin 2 mediates appetite suppression during pancreatic cancer cachexia. Nat Commun.

[40] Naudé PJ, Eisel UL, Comijs HC (2013). Neutrophil gelatinase-associated lipocalin: a novel inflammatory marker associated with late-life depression. J Psychosom Res.

[41] Mishra J, Dent C, Tarabishi R (2005). Neutrophil gelatinase-associated lipocalin (NGAL) as a biomarker for acute renal injury after cardiac surgery. Lancet.

[42] Wang G, Weng YC, Chiang IC (2020). Neutralization of lipocalin-2 diminishes stroke-reperfusion injury. Int J Mol Sci.

[43] Mucha M, Skrzypiec AE, Schiavon E (2011). Lipocalin-2 controls neuronal excitability and anxiety by regulating dendritic spine formation and maturation. Proc Natl Acad Sci U S A.

[44] Xiao Y, Xu A, Hui X (2013). Circulating lipocalin-2 and retinol-binding protein 4 are associated with intima-media thickness and subclinical atherosclerosis in patients with type 2 diabetes. PLoS One.

[45] Dekens DW, Eisel U, Gouweleeuw L (2021). Lipocalin 2 as a link between ageing, risk factor conditions and age-related brain diseases. Ageing Res Rev.

