# Supplementary material for: Higher serum lipocalin 2 is associated with post-stroke depression at discharge
Source: BMC Neurol. 2023 Aug 5;23:294. doi: 10.1186/s12883-023-03319-y (PMC10403916; doi:10.1186/s12883-023-03319-y)
Supplement: Supplementary file 1 — Additional file 1. Relationship between serum LCN2 and gender, age. Relationship between serum LCN2 and Stroke subtypes. [file 12883_2023_3319_MOESM1_ESM.docx]

**Relationship between serum LCN2 and gender, age**

We found slightly higher serum LCN2 levels in females than in males (128.34±41.28 vs. 121.54±40.87), but it was not statistically significant (*P*=0.119). Age was positively correlated with serum LCN2 levels (r=0.135, *P*=0.010).


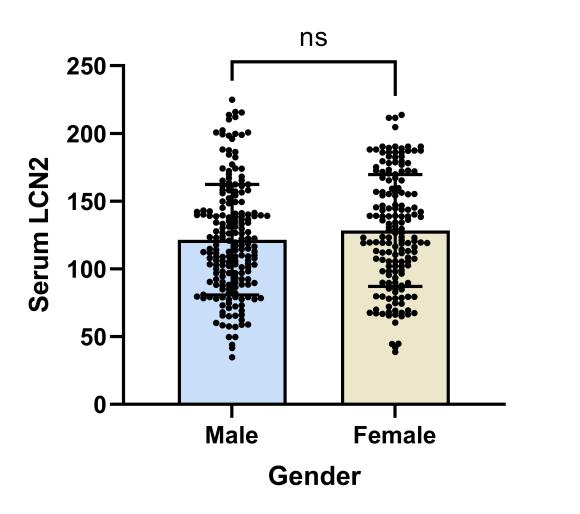

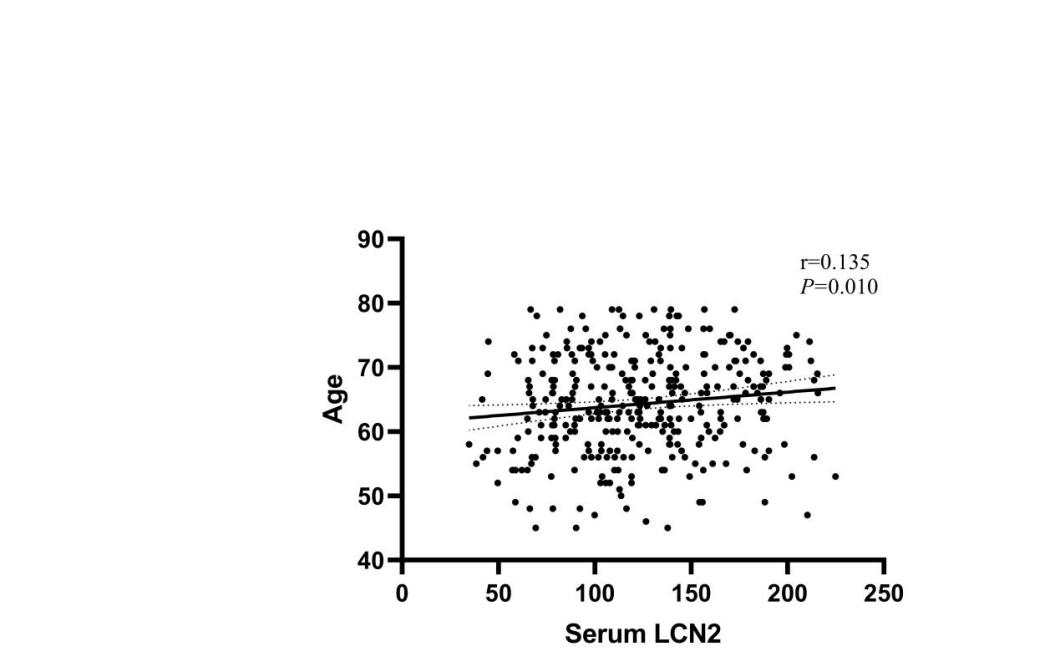


Abbreviations: *LCN2* Lipocalin 2, *ns* no significance.

**Relationship between serum LCN2 and Stroke subtypes**

Comparing serum LCN2 levels among different stroke subtypes, P-values were adjusted according to Bonferroni correction methods. Only the difference between LAA group and SAO group was statistically significant (*P*<0.01), and no statistically significant difference was found in pairwise comparison among other groups (*P*>0.05).


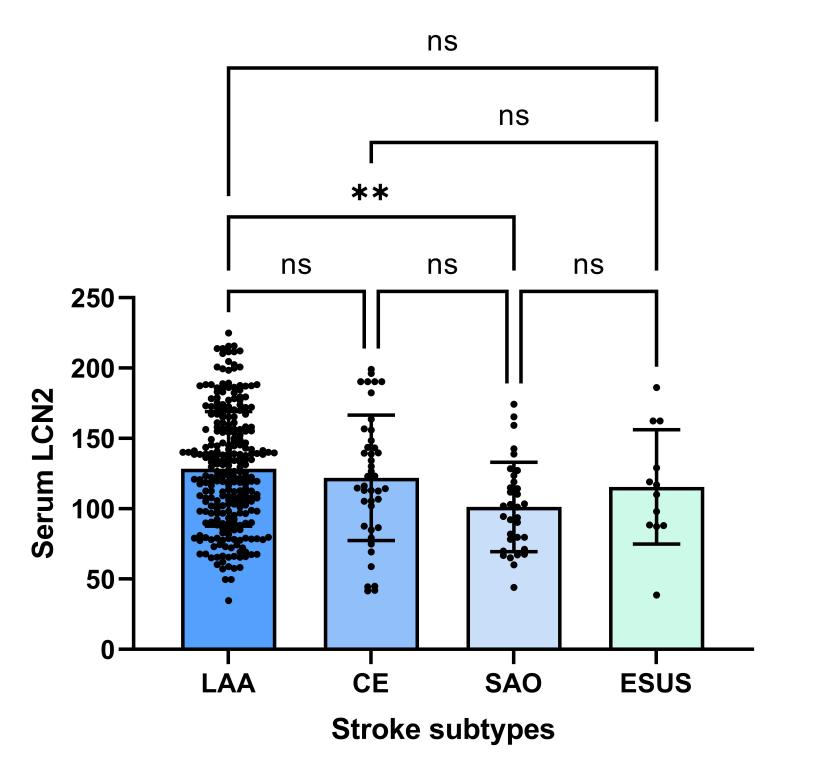


Abbreviations: *LCN2* Lipocalin 2, *LAA* Large artery atherosclerosis, *CE* Cardioembolism, *SAO* small-artery occlusion, *ESUS* embolic stroke of undetermined source, **** *P*<0.01, *ns* no significance.
